# The Role of Glutamatergic Gene Polymorphisms in the Clinical Phenotypes of Schizophrenia

**DOI:** 10.3390/genes14030575

**Published:** 2023-02-24

**Authors:** Evgeniya G. Poltavskaya, Elena G. Kornetova, Maxim B. Freidin, Ivan V. Pozhidaev, Diana Z. Paderina, Anna V. Bocharova, Arkadiy V. Semke, Nikolay A. Bokhan, Svetlana A. Ivanova, Olga Y. Fedorenko

**Affiliations:** 1 Mental Health Research Institute, Tomsk National Research Medical Center of the Russian Academy of Sciences, 634014 Tomsk, Russia; 2 Department of Psychiatry, Addictology and Psychotherapy, Siberian State Medical University, 634050 Tomsk, Russia; 3 Research Institute of Medical Genetics, Tomsk National Research Medical Center of the Russian Academy of Sciences, 634050 Tomsk, Russia; 4 School of Biological and Behavioural Sciences, Queen Mary University of London, London E1 4NS, UK

**Keywords:** schizophrenia, clinical heterogeneity, polymorphism, *GRIN2A*, *GRIN2B*, *SLC1A2*, *SLC17A7*, *GRM3*, *GRM7*, *GRM8*

## Abstract

Background: Personal variations in genetic risk for schizophrenia relate to its phenotypic heterogeneity—both in disorder development and clinical manifestations. Abnormal glutamatergic neurotransmitter system functioning is integrated in the pathogenesis of schizophrenia. Methods: A sample of 805 Russian schizophrenia patients from the Siberian Federal region was investigated. We examined the association of 39 single nucleotide polymorphisms in eight genes (*GRIN2A*, *GRIN2B*, *SLC1A2*, *SLC1A3*, *SLC17A7*, *GRM3*, *GRM7*, and *GRM8*) involved in the glutamatergic system with the development of clinical heterogeneity of schizophrenia. The MassARRAY Analyzer 4 was used for genotyping. Results: *GRIN2A* rs11644461, rs8057394 and *GRIN2B* rs7313149 are associated with the continuous type of schizophrenia. The *GRIN2A* rs8057394*G allele is a relative risk factor (*p* = 0.019) for developing the continuous type of schizophrenia. We found a nominally significant association between negative symptoms of schizophrenia and *SLC17A7* rs62126236. The *SLC17A7* rs62126236*T allele has a protective effect (*p* = 0.039) against predominant negative symptoms in schizophrenia. The total Positive and Negative Syndrome Scale (PANSS) scores were significantly associated with *GRIN2A* rs9788936 after adjusting for multiple testing (*p* = 0.001). Conclusions: In this study the contribution of the glutamatergic gene polymorphisms to the clinical heterogeneity of schizophrenia has been demonstrated.

## 1. Introduction

Dopaminergic and glutamatergic abnormalities are leading hypotheses in understanding schizophrenia [1,2,3]. Glutamate is the most abundant excitatory amino acid neurotransmitter, activating G protein-coupled metabotropic receptors mediating slow synaptic transmission and ionotropic receptors mediating fast synaptic transmission in the brain [4]. Altered excitatory signaling, via a hypofunction of the N-methyl-D-aspartate (NMDA)-type glutamate receptor (NMDAR), is considered a key contributor to the schizophrenia disease process [5]. The NMDAR non-competitive antagonists, such as phencyclidine, dizocilpine and ketamine, are well known to provoke positive and negative schizophrenia-like symptomatology in otherwise healthy people [6] as well as inducing relapse in schizophrenia patients [7]. At the same time, ketamine’s action at the NMDAR can result in dose-dependent antidepressant responses in humans [8].

GluN2A, encoded by *GRIN2A* (glutamate receptor, ionotropic, N-methyl-D-aspartate 2A), is the highest expressed among NMDAR GluN2 subunits in the human CNS [9]. The variable (GT)n polymorphism located in the promoter region of *GRIN2A* was shown to be related to schizophrenia. Moreover, this (GT)n repeat could impact the severity of chronic outcomes in a length-dependent manner [1,10]. Recently we demonstrated *GRIN2A* rs7206256 to be linked to the early manifestation of this disease [11]. Moreover, *GRIN2A* rs7192557 contributes to the development of antipsychotic induced limb-truncal tardive dyskinesia in patients with schizophrenia [12].

The *GRIN2B* (glutamate receptor, ionotropic, N-methyl-D-aspartate 2B) gene, encoding the NMDAR NR2B subunits, is crucial in corticogenesis and brain plasticity. Moreover, reported associations of *GRIN2B* polymorphisms and schizophrenia have been published [13]. Recently, we demonstrated an association of the *GRIN2B* rs7313149 with the early manifestation of schizophrenia [11].

Excitatory amino acid transporters (EAATs) in addition to NMDAR have drawn considerable attention due to their direct influence on glutamatergic neurotransmission by reuptake of glutamate excess from the synaptic cleft. Of these, EAAT2 (also known as solute carrier family 1, member 2 encoded by the *SLC1A2* gene) has been extensively investigated in schizophrenia pathogenesis. Previously, a haplotype association between *SLC1A2* polymorphisms and schizophrenia in Japanese patients was reported [14]. However, this association was not confirmed in a study on a larger sample [15]. An association of *SLC1A2* rs12360706 with schizophrenia was found in the Chinese population, and heterozygotes had a higher proportion of psychosis in their family history [16].

EAAT2 is the major glutamate reuptake mechanism. Furthermore it contributes to the brain’s energy metabolism through glutamate transport into astrocytes for its further participation in the glutamate-glutamine cycle. EAAT2-mediated glutamate uptake is crucial for normal oligodendrocyte function, since its inhibition results in demyelination, axonal damage, and cell death. Abnormal EAAT2 expression has been found in multiple neuropsychiatric diseases. Several teams of researchers investigated altered EAAT2 in people with schizophrenia, reporting reduced expression in the dorsolateral prefrontal cortex and in the parahippocampal region. In addition, carriers of a risk haplotype of the metabotropic glutamate receptor 3 (*GRM3*) for schizophrenia had lower EAAT2 expression in the prefrontal cortex as well as impaired cognitive function in respect of verbal fluency and list learning [17]. Such abnormal glutamatergic transmission may play a fundamental role in working memory deficits observed in patients with schizophrenia and could underlie a progressive loss of grey matter throughout the brain. Studies showed associations between the *SLC1A2* polymorphism and cognitive functions in patients with schizophrenia [16,18,19]. A haplotype of the *SLC1A2* gene SNPs rs1042113, rs10768121, and rs12361171 was identified as a risk factor for tardive dyskinesia [12].

In addition to the evidence implicating postsynaptic neurotransmission and signaling in schizophrenia, there is accumulating evidence implicating the presynaptic component of glutamatergic synapses. Vesicular glutamate transporters (VGLUTs) transfer glutamate into vesicles for subsequent release into the synaptic cleft. VGLUTs modulate synaptic activity through effects on glutamate storage and release [20]. Three such VGLUTs are encoded by the solute carrier genes *SLC17A6-8*. VGLUT1 (*SLC17A7*) and VGLUT2 (*SLC17A6*) are expressed in glutamate-containing neurons, while VGLUT3 (*SLC17A8*) is expressed in some neurons using other neurotransmitters, e.g., acetylcholine or serotonin. The importance of glutamatergic vesicular transport is apparent from the effects of full knockouts of VGLUT1 and VGLUT2, which are incompatible with normal life. VGLUT1 knockout mice are found to die after weaning, while VGLUT2 knockouts die immediately after birth [21]. Changes in the expression of VGLUTs have been linked to various brain disorders along with schizophrenia, Alzheimer’s, and Parkinson’s disease [22].

The metabotropic glutamate receptors (mGluRs) are G-protein-coupled receptors. The group II metabotropic glutamate receptors, mGlu2 and mGlu3, respectively encoded by *GRM2* and *GRM3*, primarily act as presynaptic autoreceptors and are found to be involved in synaptic plasticity and brain function [23].

There have been several reports of a link between *GRM3* variants and schizophrenia [24,25]. In a family-based association study, AA homozygotes of *GRM3* hCV11245618 demonstrated poorer performance on several tests of prefrontal and hippocampal function, which are considered cognitive phenotypes for the disorder [25]. Furthermore, this SNP was also associated with reduced neuroimaging measures of synaptic neurotransmission and glutamatergic function as well as with less expression of EAAT2 in post-mortem brains [25].

A later genome-wide association study (GWAS) recognized *GRM3* as a gene containing potential schizophrenia risk variants [26]. A large meta-analysis of 14 SNPs in *GRM3* found significant associations and population specificity for three SNPs (rs2237562, rs13242038, and rs917071) [27].

The metabotropic glutamate receptor 7 (mGlu7) is a member of the group III mGlu receptors and is abundantly expressed presynaptically in both excitatory and inhibitory synapses, thereby modulating both glutamate and γ-aminobutyric acid (GABA) release. The mGlu7 is considered a therapeutic target for several psychiatric and neurological disorders, and *GRM7* polymorphisms have been linked to schizophrenia, depression, autism, and ADHD [28,29,30,31,32].

*GRM8* is a further group III mGluR and a schizophrenia candidate gene. Several studies have shown associations of *GRM8* loci in Chinese [33], Japanese [34], and Iranian [35] populations.

In the present study, we investigated the potential role of 39 single nucleotide polymorphisms of these eight glutamatergic genes, chosen on the basis of the previous studies reviewed above, which highlight their importance in schizophrenia, in the development of the clinical heterogeneity of the disease. This was studied in terms of the leading symptoms (negative or positive), the severity of clinical symptoms (assessed by the Positive and Negative Syndrome Scale (PANSS)), and the course (continuous or episodic) of schizophrenia.

## 2. Materials and Methods

### 2.1. Patients

Ethical legislative criteria for this work are described elsewhere (protocol N142 approved on 14 May 2021) [11]. A total of 805 Russian schizophrenia patients (ages 18–60) from four different psychiatric clinics in the Siberian region listed in our previous work were recruited [11]. The inclusion standards were a verified schizophrenia diagnosis (F20) [36] and the patient’s informed agreement. The examined patients were recruited from the Siberian Region, with Caucasian/European appearance and unrelated by blood to each other. The sample did not include patients suffering from psychoorganic disorders and somatic diseases with decompensation. The PANSS was applied for psychopathological assessment [37]. In the study group, the total PANSS score was 102 (92; 109) (median and lower-upper quartiles: Me (Q1; Q3)). The continuous or episodic course of the disorder was concluded on the basis of the fifth character of ICD-10.

We proceeded from Crow’s dichotomous concept of schizophrenia (positive and negative) for psychopathological estimation [38]. This concept postulates two “pathological aspects” underlying schizophrenia: a positive component (potentially sensitive to antipsychotics) and a negative component (often progressive and associated with a deficit state and poor long-term outcome). To investigate the involvement of the studied genetic variants in the progression of predominant negative or positive symptoms according to the PANSS survey data, the initial sample of 805 schizophrenia patients was split into 2 subgroups: a subgroup of 391 patients with predominant negative symptoms (PANSS positive scale score 20 (17; 24), PANSS negative scale score 27 (24; 31)) and a subgroup of 414 patients with predominant positive symptoms (PANSS positive scale score 27 (23; 30), PANSS negative scale score 24 (21; 27)). The remaining patients had mixed symptoms, without predominance of positive or negative symptoms, and were excluded from the comparison. To analyze the associations of the studied genetic variants in the course of schizophrenia, 2 subgroups were distinguished from the general group of patients with schizophrenia: 398 cases suffered continuously, and 257 patients experienced an episodic course of the disease.

### 2.2. Genetic Analysis

Venous blood samples were taken in EDTA-containing tubes followed by DNA extraction by the standard phenol-chloroform method.

Inclusion criteria for single-nucleotide polymorphisms (SNP) selection are described elsewhere [39]. Genotyping of thirty nine SNPs in *GRIN2A* (rs9989388, rs7190619, rs7196095, rs7192557, rs9788936, rs7206256, rs4782039, rs1345423, rs11644461, rs11646587, rs8057394); *GRIN2B* (rs12300851, rs220599, rs7313149, rs12827536, rs10772715, rs10845838, rs1805481, rs2192970, rs2300242); *SLC1A2* (rs3812778, rs3829280, rs1042113, rs10768121, rs11033046, rs12361171, rs3088168, rs12294045, rs10742338); *SLC1A3* (rs2229894); *SLC17A7* (rs62126236); *GRM3* (rs1468412, rs2299225); *GRM7* (rs3749380, rs17031835, rs12491620, rs1450099); *GRM8* (rs2299472, rs2237748) was carried out with the use of a SEQUENOM MassARRAY^®^ Analyzer 4 mass spectrometer (Agena Bioscience™) using the SEQUENOM Consumables iPLEX Gold 96 kit based at The Core Facility “Medical Genomics”, TNRMC, RAS.

### 2.3. Statistical Analysis

Statistical analysis was done using R 4.0.4. The Hardy–Weinberg equilibrium (HWE) of genotypic frequencies was tested using the χ2 test. Logistic or linear regression was applied to test the association between clinical phenotypes and genetic variants (additive model), while correcting for age, sex, chlorpromazine equivalent (CPZeq), and duration of disease. Bonferroni correction was applied after calculating the number of independent tests following the approach described by Li and Li [40] and after excluding SNPs that did not pass the HWE test.

## 3. Results

Details about the studied patient population are presented in Table 1.

There are well-established sex differences in the temporal incidence and prevalence of schizophrenia [41]. At a younger age, as is the case in our population, men are in excess, but at later age relatively more women present with schizophrenia than men.

Deviation from the HWE was found for *SLC1A2* rs10742338; hence, this polymorphism was excluded from further consideration (Table S1). Using the rest of the SNPs, we estimated the number of independent tests was 31; therefore, we set the significance level for the current study as 0.05/31 = 0.0016.

### 3.1. Association of Studied SNPs with the Course of Schizophrenia (Continuous vs. Episodic)

In the present study, we compared groups of schizophrenia patients with a continuous and episodic type of the course of the disease. The continuous course is considered less favorable for patients; therefore, we compared patients with a continuous course of schizophrenia with patients with an episodic type in analysis of the association with the SNPs. We found nominally significant (*p* < 0.05) associations with the continuous course of schizophrenia for the following polymorphisms: *GRIN2A* rs11644461, rs8057394; *GRIN2B* rs7313149 (Table 2). The *GRIN2A* rs8057394*G allele is a relative risk factor for developing the continuous type of schizophrenia. *GRIN2A* rs11644461*T and *GRIN2B* rs7313149*T alleles have protective effect against developing the continuous type of schizophrenia.

### 3.2. Association of Studied SNPs with Predominant (Negative vs. Positive) Symptoms of Schizophrenia

We investigated genetic features in groups of patients with a predominance of negative or positive schizophrenia symptoms. Since the progression of negative symptoms is believed to be a less favorable schizophrenia prognosis factor, we considered patients with predominant negative symptoms as the “case” group versus patients with positive symptoms as “controls” in genetic analysis of this characteristic. We found a nominally significant association between negative symptom predominance and *SLC17A7* rs62126236 polymorphism (Table 3). The results show that the *SLC17A7* rs62126236*T has a protective effect (*p* = 0.039) against predominant negative symptoms in schizophrenia.

### 3.3. Association of Studied SNPs with Intensity of Symptoms in Patients with Schizophrenia

The PANSS scale was used to assess the intensity of symptoms. The PANSS scale has several sections for assessing positive, negative, and general psychopathological symptoms. We carried out an association analysis between the total scores on the PANSS scale (for each section separately, as well as using the total score for the entire scale) and the polymorphisms in the studied genes of the glutamatergic system.

Four polymorphisms, *GRIN2A* rs9788936 (*p* = 0.010), *GRIN2A* rs11646587 (*p* = 0.036), *GRM8* rs2299472 (*p* = 0.034), and *SLC17A7* rs62126236 (*p* = 0.044), were associated with the intensity of negative symptoms according to the PANSS scores (Table 4). Also, we found an association of *GRIN2A* rs9788936 (*p* = 0.014) and *GRIN2B* rs7313149 (*p* = 0.025) with the intensity of positive symptoms (Table 4).

Comparison of totals on the PANSS scale in the block of tests for assessing general psychopathological symptoms revealed differences for the *GRIN2A* rs8057394 (*p* = 0.011), *GRIN2A* rs9788936 (*p* = 0.012), and *SLC1A2* rs12361171 (*p* = 0.027) polymorphisms.

During the study, we also assessed the total scores on the PANSS (Table 4). As a result of this analysis, associations of four polymorphisms, *GRIN2A* rs9788936 (*p* = 0.001), *GRIN2A* rs8057394 (*p* = 0.011), *GRIN2B* rs7313149 (*p* = 0.016), and *SLC1A2* rs12361171 (*p* = 0.040) with the intensity of symptoms in schizophrenia were identified.

**Figure 1:**
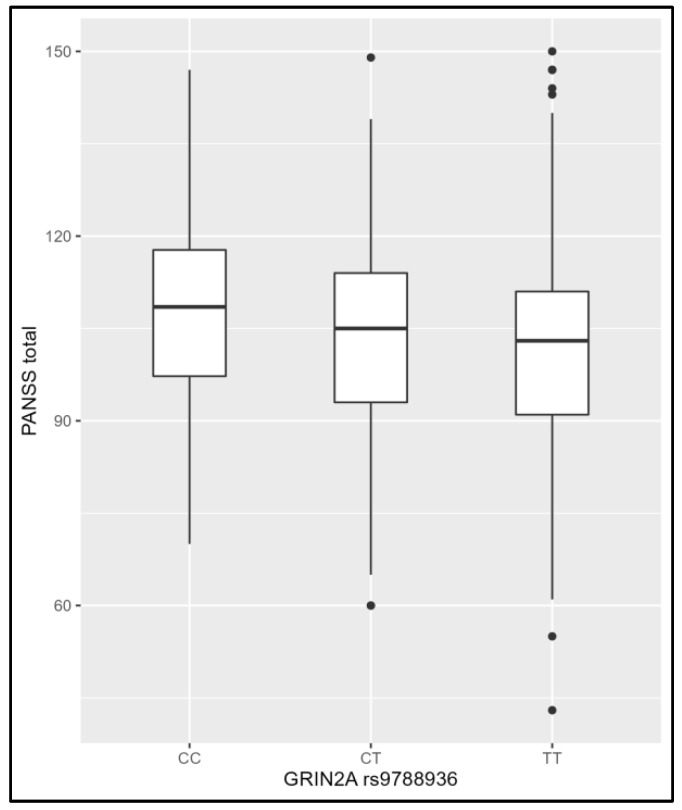
Association between *GRIN2A* rs9788936 and total scores on the Positive and Negative Syndrome Scale (PANSS).

No statistically significant associations between the SNPs and clinical phenotypes of schizophrenia were found after adjusting for multiple testing, except *GRIN2A* rs9788936 and total scores on the PANSS (Figure 1). The carrying of *GRIN2A* rs9788936*T is associated with lower total scores on the PANSS.

## 4. Discussion

Schizophrenia is a highly heterogeneous mental disorder. Its management significantly depends on whether the course is continuous or episodic. Some of the diagnostic symptoms of this disorder are known as poor prognostic factors. Persistent negative symptoms, as well as a continuous pattern of the course of the disease, are associated with a higher progression of schizophrenia.

Glutamatergic neurotransmission is widespread in the central nervous system, as basically all corticofugal and intracortical connections use this neurotransmitter [42]. NMDARs mediate a relatively slow ionotropic component of excitatory synaptic transmission. These receptors are crucial for brain development and neuroplasticity, while their hyperfunction can result in various neurodegenerative disorders mediated via calcium-mediated excitotoxicity [43]. The *GRIN2A* gene encodes the NMDAR GluN2A subunit, which plays a critical role during postnatal brain development, as its expression increases while that of the GluN2B subunit (encoded by the *GRIN2B* gene) decreases. The *GRIN2A* and *GRIN2B* genes are being extensively studied as plausible candidate genes for the susceptibility to schizophrenia and other neurodegenerative or neurodevelopmental disorders. We previously showed that the *GRIN2A* and *GRIN2B* genes are associated with early onset of schizophrenia [11].

The *SLC1A2* gene encodes EAAT2, which is responsible for removal of glutamate from the synaptic cleft. *SLC1A2* has been implicated in several neurological and psychiatric conditions, including schizophrenia, autism, and bipolar disorder [44]. We previously showed that the *PIP5K2A* gene, whose dysfunction affects the functioning of glutamate transporters [45], is also associated with the course of schizophrenia [46].

The *GRM8* gene encodes a presynaptic metabotropic glutamate receptor 8 (mGluR8), modulating neuronal excitability by inhibiting glutamate release at the synapse. Previously, *GRM8* rs2299472*CC was shown to be associated with schizophrenia in the Uygur Chinese population [33]. We could find an association of *GRM8* rs2299472 with the intensity of negative symptoms according to the PANSS scores. In our study, schizophrenia patient carriers of the *GRM8* rs2299472*C allele had lower scores on the Negative Subscale of PANSS.

The association between *GRIN2A* rs9788936 and total scores on the PANSS survived multiple tests and is likely to be a replicable finding.

It is worth noting that other important SNPs in glutamatergic system genes were omitted because of limited screening criteria but deserve further investigation in our sample. For example, in a Chinese sample, rs12360706 in *SLC1A2* was recently found to be associated with schizophrenia, and heterozygotes had a higher proportion of psychosis in their family history [16]. Moreover, three further SNPs from a GWAS study of *GRM3* mentioned previously should also be investigated [27].

In the present study, we were able to detect the contribution of the *GRIN2A*, *GRIN2B*, *GRM8*, *SLC1A2,* and *SLC17A7* genes of the glutamatergic system to determining the clinical heterogeneity of schizophrenia, which is important for the prognosis and outcome of the disease. The results may be used as predictors of the adverse course of schizophrenia, overall predominance of negative symptoms, and disruption of integrity, which is a contribution to precision medicine. Nevertheless, the identified associations still need to be explored in much greater depth in larger cohorts and other populations.

### Limitations

The study design was cross-sectional, and attempts to find lifetime worst occasion symptoms were therefore not possible. Symptoms were assessed during the diagnostic interview and could therefore be likely to reflect the effect of medication, since the studied patients were not drug free; thus, we cannot distinguish the relationship of genotype to the underlying symptom profile from its relationship with treatment response. Longitudinal studies of initially drug-naïve patients would be needed to overcome this limitation. The fact that only Caucasians were included limits the study in terms of generalization, although we tried to achieve ethnic homogeneity of the sample. Despite the relatively good sample size, genetic correlations were modest and did not survive multiple testing corrections, except *GRIN2A* rs9788936 and total scores on the PANSS. While limited resources prevented a comprehensive study of all potentially functional or marker SNPs in all genes related to glutamatergic synaptic function, we chose to genotype a series of previously studied SNPs in genes that our work and that of others have strongly implicated in schizophrenia. As mentioned above, this study did not include all potentially associated SNPs. As other neurotransmitter systems in addition to glutamate are implicated in the pathology of schizophrenia, focusing only on the glutamatergic system could be a source of bias. Further studies would ideally involve GABAergic and dopaminergic genes and their potential interactions with our current findings.

## 5. Conclusions

This study indicates the likely contribution of the *GRIN2A*, *GRIN2B*, *GRM8*, *SLC1A2*, and *SLC17A7* genes to the development of clinical heterogeneity in schizophrenia. Associations of these genes with the course, the predominant symptoms, as well as the intensity of symptoms in schizophrenia were identified. Thus, we have shown that the genes of the glutamatergic system can contribute to determining the nature of the course of schizophrenia, thereby determining individual prospects following the development of the disease.

## Figures and Tables

**Table 1:** Demographic and clinical parameters of the studied patients.

Sample Size, n	805
Sex, n (%)	Men: 423 (52.5%) Women: 382 (47.5%)
Age, years, Me (Q1; Q3)	38 (32; 49)
Duration of illness, years, Me (Q1; Q3)	13 (7; 22)

n—number of patients; Me—Median; Q1—Quartile 1; Q3—Quartile 3.

**Table 2:** Odds ratios calculated for polymorphisms of the studied genes associated with the type of course of schizophrenia.

Gene	SNP	Effect Allele	Other Allele	OR	Lower 95%	Upper 95%	*p*-Value
*GRIN2A*	rs11644461	T	C	0.739	0.576	0.950	0.018
*GRIN2A*	rs8057394	G	C	1.361	1.051	1.763	0.019
*GRIN2B*	rs7313149	T	C	0.742	0.560	0.984	0.038

Note: SNP—Single Nucleotide Polymorphism. Analysis was carried out using logistic regression. Disease type (continuous vs. episodic) was used as a response variable; genotypes (encoded 0, 1, 2) were used as predictors. Adjustment was done for age, sex, chlorpromazine equivalent, and duration of disease. Odds ratios (OR) and 95% confidence intervals are provided for results with at least nominal statistical significance (*p* < 0.05).

**Table 3:** Odds ratios calculated for polymorphisms of the studied genes associated with the predominant symptoms of schizophrenia (negative or positive).

Gene	SNP	Effect Allele	Other Allele	OR	Lower 95%	Upper 95%	*p*-Value
*SLC17A7*	rs62126236	T	C	0.778	0.613	0.988	0.039

Note: SNP—Single Nucleotide Polymorphism. Analysis was carried out using logistic regression. Disease symptoms (negative vs. positive) were used as a response variable; genotypes (encoded 0, 1, 2) were used as predictors. Adjustment was done for age, sex, chlorpromazine equivalent (CPZeq), and duration of disease. Odds ratios (OR) and 95% confidence intervals are provided for results with at least nominal statistical significance (*p* < 0.05).

**Table 4:** Results of analysis of association between PANSS scores and genetic variants.

Gene	SNP	Effect Allele	Other Allele	Estimate	SE	t Value	*p*-Value
Association between PANSS N1-7 and genetic variants							
*GRIN2A*	rs9788936	T	C	−0.159	0.061	−2.592	0.010
*GRIN2A*	rs11646587	G	A	0.123	0.059	2.106	0.036
*GRM8*	rs2299472	C	A	−0.114	0.053	−2.122	0.034
*SLC17A7*	rs62126236	T	C	−0.115	0.057	−2.018	0.044
Association between PANSS P1-7 and genetic variants							
*GRIN2A*	rs9788936	T	C	−0.151	0.062	−2.454	0.014
*GRIN2B*	rs7313149	T	C	−0.133	0.059	−2.248	0.025
Association between PANSS G1-16 and genetic variants							
*GRIN2A*	rs8057394	T	C	0.139	0.055	2.536	0.011
*GRIN2A*	rs9788936	T	C	−0.155	0.062	−2.505	0.012
*SLC1A2*	rs12361171	T	A	0.113	0.051	2.209	0.027
Association between PANSS total and genetic variants							
*GRIN2A*	rs9788936	T	C	−0.196	0.061	−3.190	0.001
*GRIN2A*	rs8057394	T	C	0.138	0.054	2.534	0.011
*GRIN2B*	rs7313149	T	C	−0.140	0.058	−2.408	0.016
*SLC1A2*	rs12361171	T	A	0.104	0.051	2.058	0.040

Note: PANSS—Positive and Negative Syndrome Scale; N1-7—Negative Subscale containing 7 items; P1-7—Positive Subscale containing 7 items; G1-16—General Psychopatology Subscale containing 16 items; SNP—Single Nucleotide Polymorphism; SE—Standard Error. Analysis was carried out using multiple regression. PANSS was used as a response variable; genotypes (encoded 0, 1, 2) were used as predictors. Prior to the analysis, PANSS distribution was transformed to standard normal distribution N [0, 1] using quantile normalization. Adjustment was done for age, sex, CPZeq, and duration of disease. Regression coefficients and standard errors are provided for results with at least nominal statistical significance (*p* < 0.05).

## Data Availability

The datasets generated for this work will not be made publicly accessible, although they are available on reasonable request to Olga Yu. Fedorenko (f_o_y@mail.ru), following approval of the Board of Directors of the MHRI, in line with local guidelines and regulations.

## Supplementary Materials

The following supporting information can be downloaded at: https://www.mdpi.com/article/10.3390/genes14030575/s1, Table S1: The basic information of analyzed polymorphic variants.
