# Supplementary material for: The Role of Glutamatergic Gene Polymorphisms in the Clinical Phenotypes of Schizophrenia
Source: Genes (Basel). 2023 Feb 24;14(3):575. doi: 10.3390/genes14030575 (PMC10048659; doi:10.3390/genes14030575)
Supplement: Supplementary file 1 [file genes-14-00575-s001.zip › genes-2229008-supplementary.pdf]

Table S1. The basic information of analyzed polymorphic variants.

Gene	SNP	Chromosome: Location	Location Region	Alleles	MAF	χ^2	p-Value
GRIN2A	rs9989388	16:9872282	intron variant	C/T	0.19 (T)	0.255	0.614
	rs7190619	16:9985267	intron variant	G/A	0.07 (A)	0.665	0.415
	rs7196095	16:9791975	intron variant	T/C/G	0.30 (C)	0.110	0.740
	rs7192557	16:10029612	intron variant	G/A/C/T	0.32 (A)	0.280	0.597
	rs9788936	16:10011603	intron variant	T/C	0.24 (C)	0.014	0.906
	rs7206256	16:10103066	intron variant	A/G/T	0.44 (G)	0.417	0.518
	rs4782039	16:9913110	intron variant	T/C	0.22 (C)	0.659	0.417
	rs1345423	16:10154207	intron variant	G/A/C/T	0.32 (G)	0.241	0.624
	rs11644461	16:10027033	intron variant	T/C	0.23 (C)	0.686	0.408
	rs11646587	16:9779462	intron variant	G/A	0.29 (A)	0.373	0.541
	rs8057394	16:10021631	intron variant	C/G	0.43 (C)	0.354	0.552
GRIN2B	rs12300851	12:13815471	intron variant	T/A/C	0.10 (C)	0.066	0.798
	rs220599	12:13822364	intron variant	G/A	0.44 (A)	2.664	0.103
	rs7313149	12:13675353	intron variant	T/A/C/G	0.21 (C)	1.875	0.171
	rs12827536	12:13943223	intron variant	C/T	0.22 (T)	0.912	0.340
	rs10772715	12:13885069	intron variant	G/A	0.43 (A)	0.006	0.938
	rs10845838	12:13741462	intron variant	G/A	0.38 (A)	0.482	0.487
	rs1805481	12:13610521	intron variant	A/C	0.43 (C)	0.832	0.362
	rs2192970	12:13683379	intron variant	G/A	0.11 (A)	0.195	0.659
	rs2300242	12:13687363	intron variant	A/T	0.48 (T)	0.604	0.437
SLC1A2	rs3812778	11:35255723	3 prime UTR variant	G/A	0.10 (A)	2.547	0.110
	rs3829280	11:35255176	3 prime UTR variant	A/C/T	0.13 (T)	2.916	0.088
	rs1042113	11:35286822	synonymous variant	T/C	0.23 (C)	0.081	0.777
	rs10768121	11:35258109	3 prime UTR variant	A/C/G	0.35 (C)	1.411	0.235
	rs11033046	11:35253386	3 prime UTR variant	T/A	0.36 (A)	0.763	0.382
	rs12361171	11:35256786	3 prime UTR variant	T/A/C	0.36 (A)	2.575	0.109
	rs3088168	11:35251721	3 prime UTR variant	T/C	0.36 (C)	0.778	0.378
	rs12294045	11:35257754	3 prime UTR variant	C/G/T	0.20 (T)	0.015	0.904
	rs10742338	11:35255541	3 prime UTR variant	T/A/C	0.10 (T)	4.312	0.038 *
SLC1A3	rs2229894	5:36686302	3 prime UTR variant	G/A/C	0.43	0.552	0.457
SLC17A7	rs62126236	19:49441696	intron variant	T/C	0.19 (C)	0.458	0.499
GRM3	rs1468412	7:86804135	intron variant	A/T	0.38 (T)	0	1
	rs2299225	7:86818264	intron variant	T/G	0.03 (G)	1.247	0.264
GRM7	rs3749380	3:6861610	missense variant	C/G/T	0.42 (T)	0.299	0.585
	rs17031835	3:6880071	intron variant	C/T	0.10 (T)	0.039	0.844
	rs12491620	3:7352646	intron variant	C/G	0.18 (G)	3.215	0.073
	rs1450099	3:7496689	intron variant	T/G	0.38 (T)	0.099	0.752
GRM8	rs2299472	7:126580415	intron variant	C/A/G	0.32 (A)	0.062	0.803
	rs2237748	7:126638809	intron variant	C/T	0.32 (T)	0	1

Notes: MAF—minor allele frequency; HWE $\chi 2$ and HWE p—chi-square and p-value statistics, respectively, to test the frequency distribution according to the Hardy–Weinberg equilibrium. *: significant at $p < 0.05$.
